# Standardized Forearm Angiography Increases Procedural Success Rates of Coronary Angiography and PCI: A Retrospective Analysis of an all-Comers Patient Cohort in a Real-Life Scenario

**DOI:** 10.26502/fccm.92920250

**Published:** 2022-04-07

**Authors:** Tobias Roeschl, Anas M Jano, Franziska Fochler, Mona M Grewe, Marlis Wacker, Kirstin Meier, Christian Schmidt, Lars Maier, Peter H Grewe

**Affiliations:** 1 Clinic of Cardiology and Angiology, Klinikum Neumarkt, Neumarkt, Germany; 2 Department of Internal Medicine II, University Hospital Regensburg, Germany

**Keywords:** Access side complication prevention, Aoronary forearm access, Standardized forearm angiography, Transulnar-access, Transradial-access

## Abstract

**Background:**

There is a consensus, that Transradial-Access (TRA) for coronary procedures should be preferred over Transfemoral-Access (TFA). Previously, Forearm-Artery-Angiography (FA) was mainly performed when difficulties during the advancement of the guidewire/-catheter were encountered. We explored the implication of a Standardized Forearm-Angiography (SFA) on procedural success rates of TRA under real-world conditions.

**Methods:**

In a single-center study, an all-comers-cohort of 1191 consecutive cases during 1/2020–12/2020 were assessed retrospectively. Primary TFA rates, crossover to TFA, reasons for Forearm-Artery-Access (FAA) failure, the prevalence of kinking at the level of the forearm and the occurrence of vascular complications were analyzed. Major forearm side branches including the common interosseus artery were assessed via SFA.

**Results:**

In 1191 consecutive procedures, primary FAA access was attempted in 97.9% of cases. Crossover to TFA after a primary or secondary FAA attempt was necessary in 2.8%. Severe kinking was the most frequent cause of FAA failure and occurred in 3.0% of attempts. A second or third FAA attempt to avoid TFA was successful in 81%. Severe kinking at the level of the forearm was reported in 1.8% of procedures.

**Conclusion:**

This is the first study to provide detailed success rates of a primary FAA strategy combined with a Standardized-Forearm-Angiography (SFA) in an all-comers-cohort. While severe kinking proved to be a rare but relevant challenge for FAA success, the prevalence of arterial spasm was marginal. Multiple attempts of FAA to avoid TFA might be safe possibly due to collateral blood supply by the common interosseus artery.

## 1. Introduction

Transradial-Access (TRA) has evolved from an experimental approach to standard of care for cardiac catheterization within the last 30 years [1–3]. TRA has demonstrated a lower incidence of local bleeding complications compared to transfemoral-access (TFA) and transbrachial-access. Positive outcomes of multiple randomized controlled trials comparing procedural safety of TRA vs. TFA lead to an upgrade of the recommendation for TRA to IA in recent ESC guidelines [4, 5]. Furthermore, TRA was associated with a lower risk of cardiac death and all-cause mortality after 30 days of follow-up [5, 6]. Results from the SWEDEHEART-registry suggest that primary PCI by TRA rather than TFA was associated with a lower incidence of cardiogenic shock in patients with ST-elevation myocardial infarction5. During in-depth examination of study protocols and study appendices, it became evident, that the benefits of TRA were demonstrated in highly selected study populations [7–9]. There are numerous reasons for failure to perform cardiac catheterization via TRA in general. Among these are failure of arterial cannulation, failed guidewire placement after arterial puncture and unsuccessful sheath insertion. Even after primary successful sheath insertion, arterial spasms, atherosclerotic or stenosed arteries, arterial loops at the brachioradial junction or severe tortuosity of the brachiocephalic trunk can hinder guidewire or guide catheter advancement into the coronary ostia. Therefore, these anatomical obstacles to FAA as well as operator experience, highly affect procedural success rates [10]. We believe that a precise assessment of anatomy, pathology and anatomic variants of forearm arteries is indispensable before and during cardiac catherization [11]. Under study conditions, forearm artery angiography (FA) was associated with a significantly higher procedural success rate of TRA and significantly less complications with selected patient populations [12, 13]. To investigate the importance and the feasibility of FA in daily practice and to avoid patient preselection, we retrospectively analyzed all cases of cardiac catheterization at our facility between 1/2020–12/2020. Our aim was to investigate whether a SFA had a positive impact on procedural success in an all-comers cohort. Moreover, anatomical variants of forearm arteries possibly impeding procedural success were analyzed angiographically.

## 2. Methods

In a retrospective, single-center analysis, 1191 consecutive Coronary Angiographies (CA) with or without PCI, corresponding to 940 individual patients, between 1/2020–12/2020 were analyzed. Cases who required simultaneous right and left heart catheterization were excluded only if this coincided with primary transfemoral access (n=8). Otherwise, no cases were excluded in the period under review. In our standardized approach, operating physicians were urged to attempt Forearm Artery Access (FAA) for cardiac catheterization whenever possible. Ultrasound-guided arterial puncture followed by a SFA including the ipsilateral distal brachial artery was mandatory after sheath insertion. The choice of forearm artery and side used in an individual patient was at the discretion of the operator. By institutional policy, left FAA was mandatory in patients with left internal mammary bypass graft. Arterial access sites included the distal radial artery in the anatomical snuffbox, the proximal radial artery, the ulnar artery, and the anterior interosseous artery. Primary TFA was only performed if inaccessibility of all major forearm arteries was evident from patient history or previous angiographies (e.g. dialysis shunts on both forearms). In case of failure to complete cardiac catheterization via the primarily chosen forearm artery, operating physicians were principally required to crossover to another artery on the ipsilateral or contralateral forearm by institutional policy (“forearm-only-strategy”). During the study period, cardiac catheterization was performed by seven different interventional cardiologists with at least 300 ultrasound-guided forearm artery cannulations.

### 2.1. Standardized forearm angiography (SFA)

Setup for conventional SFA was achieved by forearm positioning on a carbon fiber board (Starboard, Fa. Adept Medical Ltd., Auckland, New Zealand). A 0.8×50mm G21 needle (Braun Melsungen, Germany) was used to puncture a forearm artery under out-of-plane ultrasound guidance (9 L probe Vivid T9 GE Healthcare,Boston, Massachusetts). A 0.021-inch, 45-cm wire was then advanced into the artery lumen, and a 70-mm hydrophilic coated introducer sheath (Glidesheath Slender Terumo, 6-French or 7-French) was placed. After sheath insertion 2 mg of verapamil or 0.2 mg of nitroglycerine and 5000 IU of heparin were given through the sheath. After successful arterial cannulation of a forearm artery and subsequent sheath insertion, an angiography of the distal upper arm and the forearm up to the wrist was obtained. The distal brachial artery, the radial artery and the ulnar artery with their respective side branches were assessed. To avoid radiation exposure to visceral organs, the operating table was angled inwards by 30 degrees away from the radiation beam. Between 5 to 10 cc of contrast gent (Ultravist-370, Bayer, Leverkusen, Germany) were used for angiography. Angiographic projections were LAO 30 for right forearm angiography and PA for left FA, respectively. All fluoroscopic studies of the forearm were performed with a pulse rate of 7.5 frames per second. During contrast dye injection, the catheterization table was moved distally to follow the contrast medium column down to the wrist with a 21-cm image intensifier (AlluraClarity, Philips) to fully capture the region of interest. Radiological anatomy of arteries was assessed visually. Stenosis, loops, high origins, branching angles, and arterial tortuosity of the radial artery at the level of the brachioradial junction and of the ulnar artery at the level of the wrist were evaluated. To further classify tortuosity, loops and abnormal branching angles with respect to clinical relevance, these particular abnormalities were summarized on their respective effect on guidewire advancement during the primary FAA attempt, hereinafter referred to as “kinking”. The following classification of kinking, in three distinct degrees of severity, is based on the passableness of the aforementioned vessel sections with a guidewire rather than a visual description of anatomic variants previously reported by others [15–17].

- No kinking: Angiographically normal vessel without difficulties to advance a standard 0.035” J guidewire (Angiodyn, Braun). facilitated with leftward rotation of the head and deep inspiration when necessary.
- 1st degree kinking: Tortuous or calcified vessel which could only be passed with a hydrophilic 0.035” guidewire (Radifocus Guidewire M, Terumo) or a 0.018” guidewire (Advantage, Terumo).
- 2nd degree kinking: Severely tortuous or calcified vessel which could not be overcome with any guidewire or guide catheter handling was severely impaired resulting in termination of the procedure and crossover to another access site.

Angiographic images of vessel sections, which were difficult to pass with a standard guidewire were displayed on a reference screen to facilitate guidewire advancement. We retrospectively studied the primary TFA rate, the crossover rate to TFA, reasons for FAA failure, the prevalence of kinking at the level of the forearm and the feasibility of a forearm-only-strategy with respect to potential vascular damage and acute hand ischemia. FAA was classified as “failed” if CA or PCI was not completed via the initially chosen forearm artery. The occurrence of acute hand ischemia was monitored clinically until discharge from hospital. Patients, who had vascular complications underwent doppler sonographic assessment of the previously damaged forearm artery before discharge. Arterial flow was considered normal if doppler waveforms were at least biphasic. For statistical analysis, continuous variables were summarized as mean ± Standard Deviation (SD) and medians ± interquartile ranges ([IQR]) for non-normally distributed variables. QQ-plots and the Shapiro-Wilk test were used to assess normality. Rates of interest were reported with 95% confidence intervals respectively. There wereno missing values regarding the variables of interest. All statistical analyses were carried out with Python 3.9 and R 4.0.3. Ethics committee approval was waived for this retrospective analysis.

## 3. Results

**Figure 1:**
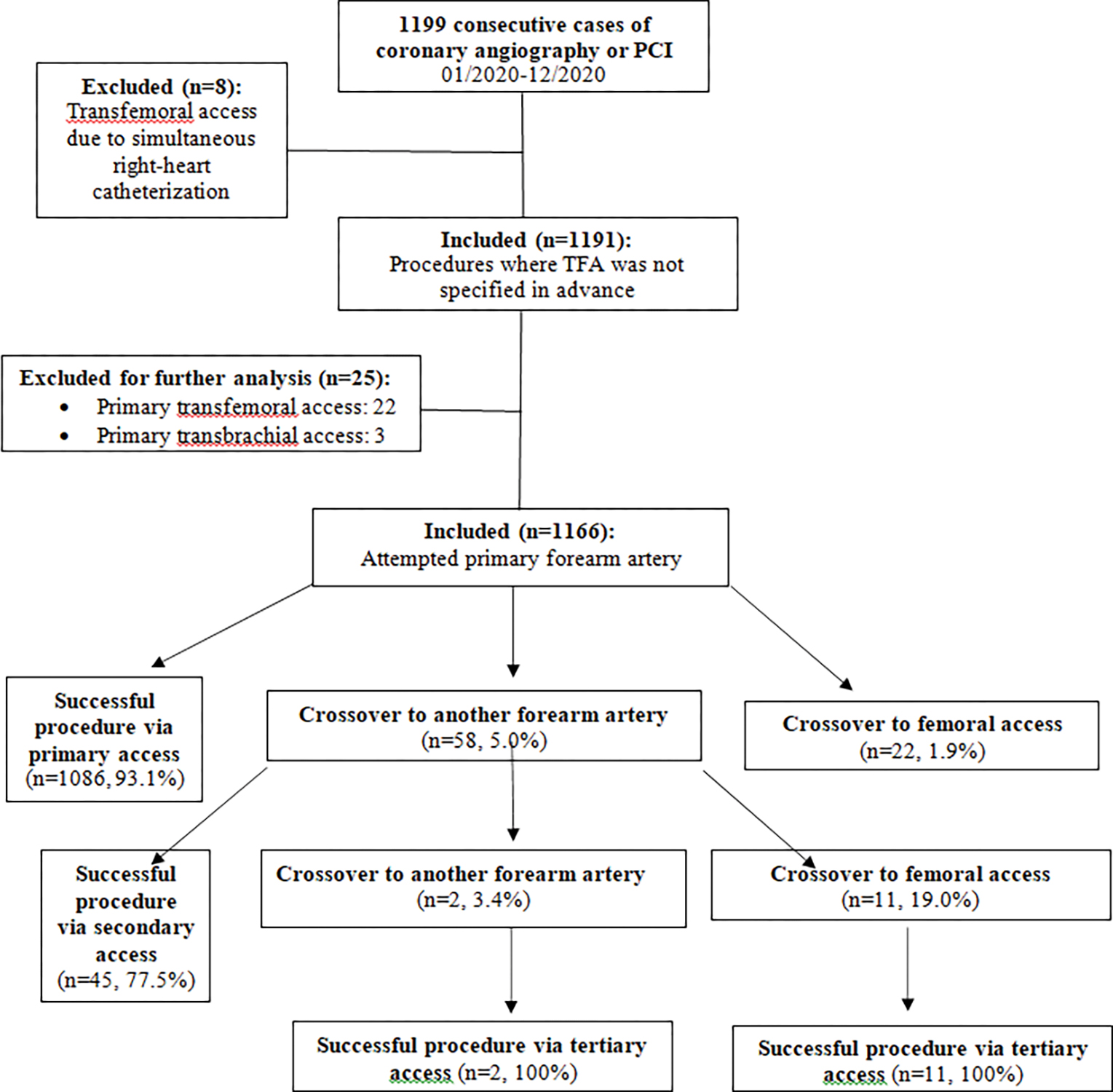
Flowchart of the exclusion process and vascular access sites.

A total of 1199 cases of cardiac catheterization were performed in our facility between 01/01/2020–12/31/2020. Eight cases were excluded as described above. In 22 (1.8%, [95% CI: 1.1% to 2.6%]) procedures, a primary femoral access and in 0.3% a primary brachial access was chosen. The reasons for primary TFA were, among others: bilateral severe kinking or severe bilateral atherosclerosis of forearm arteries known from previous angiographies (n=5), ongoing cardiopulmonary resuscitation, dialysis shunts on both forearms and planned left internal mammary artery (LIMA) graft angiography after previous left radial artery harvest and severe atherosclerosis of the left ulnar artery at the same time. The remaining 1166 procedures (97.9%), in which primary FAA was attempted, were further analyzed (Figure 1). Left FAA was chosen in 632 (54.2%) of cases and right FAA was chosen in 534 (45.8%) of cases. The relative proportions of FAA sites are summarized in Table 1. In our quasi-all-comers trial, procedures consisted of diagnostic coronary angiography in 691 (59.3%) cases and PCI in 475 (40.7%) cases. Clinical indications were suspected coronary artery disease or chronic coronary syndrome (CCS) in 959 (82.2%) cases, Acute Coronary Syndrome (ACS) in 202 (17.3%) cases and “other” in 5 (0.4%) cases (see Table 2). A total of 81 (6.9%) patients presented with STEMI. Median patient age was 71.4 [61.0, 79.4] years with a range from 25 to 95 years. 452 (38.8%) patients were above 75 years of age. Of all patients, 28.5% were female, median weight was 83.0 [72.0, 95.0] kg, mean height was 171.8 ± 9.3 cm and mean body mass index was 28.4 ± 5.1 kg/m2. 87.1% of patients had arterial hypertension, 27.9% had diabetes mellitus and 25.2% of patients were active smokers. Mean estimated glomerular filtration rate was 74.6 ± 27.9 ml/min/1.73 m2 (see Table 2). Crossover to TFA after one or more FAA attempts was observed in 2.8% [95% CI: 1.8 to 3.7%] of cases. A second or third FAA attempt at the ipsilateral or contralateral forearm was successful in 81.0% of cases, averting the potentially harmful TFA. All procedures were completed successfully. No more than three different access sites were necessary to complete the procedure in any of the procedures under review.

In 1166 cardiac catheterization sessions, a total of 1225 FAA attempts were carried out. FAA failure was observed 93 times - in 80 cases at the primarily chosen access site and in 13 cases at the secondary access site. Reasons for FAA failure were 2^nd^ degree kinking in 37 (39.8%) cases, failure to achieve arterial cannulation or failure to introduce the arterial sheath in 32 (34.4%) cases, atherosclerotic occlusion of the target vessel in 13 (14.0%) cases and other etiologies in 11 cases (11.8%). Only one instance of forearm artery spasm resulting in FAA failure was reported. Three patients had vascular access site complications leading to FAA failure. Etiologies for access failure with their respective frequencies are summarized in Table 3. During the study period, no case of acute hand ischemia or compartment syndrome was observed. In our study population, out of 1166 attempted angiographies, adequate arterial access allowing for the assessment of arterial kinking at the level of the forearm was obtained in 1142 cases. Arterial variants were classified with respect to their potential to hinder guidewire or guide catheter advancement into three separate groups: no kinking was reported in 1030 (90.2%) cases, 1st degree kinking in 91 (8.0%) and 2nd degree kinking in 21 (1.8%) cases. SFA revealed that a functional disorder (arterial spasm) was only observed in one procedure where 1st degree kinking was reported. Otherwise, 1st or 2nd degree kinking were always associated with an anatomical variant (e.g., arterial loops, arterial tortuosity, high origin of the radial artery, severe atherosclerotic burden). When the radial artery was chosen for FAA, guidewire advancement was generally hindered by an arterial loop shortly after the branching point from the brachial artery. Arterial loops were especially hard to overcome with a guidewire if the radial collateral artery or radial recurrent artery arised from the loop. This anatomical variant potentially allows for unintentional intubation of the radial collateral artery or radial recurrent artery with a standard guidewire or even with a hydrophilic 0.035” or 0.018” wire. Transulnar-artery-access (TUA) was usually impeded by a coiled course of the vessel near the wrist, aggravating arterial cannulation or due to atherosclerotic stenosis, the latter being responsible for all reported instances of TUA failure (2 out of 34 total TUA attempts). Arterial loops hindering wire passage through the ulnar artery were not observed in our study. In the middle and proximal third, the ulnar artery follows a predominantly straight course with a diameter which usually exceeds the diameter of the radial artery. During all SFA in the study period, a third forearm artery, the common interosseus artery, running parallel to the radial and ulnar artery in the interosseus membrane of the forearm, could be visualized.

## 4. Discussion

In routine clinical practice, the TFA is still used for primary arterial access to varying degrees and consistently after a failed TRA attempt despite the considerable risk of local bleeding complications. Mortality rate of retroperitoneal hemorrhage after TFA is estimated to reach up to 6.8% [14]. Multi-center trials and register studies emphasized that procedures were performed by experienced operators. However, an internationally accepted procedural approach is not yet established. Complication rates of TRA and crossover rates to TFA, described in major studies, are difficult to interpret, due to varying rates of primary TRA attempts and varying exclusion criteria for TRA [10, 15]. Clinical trials, supporting the TRA lack comparable study protocols and routine forearm angiographies were used inconsistently [12, 13]. In our retrospective study, a team of interventional cardiologists and angiologists experienced in TRA and TUA cardiac catheterization, was tracked in a real-life scenario over 12 months. Out of 1199 procedures, 8 procedures were excluded because TFA was obtained exclusively due to concomitant right heart catheterization. Previous clinical trials, performed on highly selected patients, reported TRA failure rates between 4.7–6.9% [9, 15, 16]. With female patients, TRA failure rates of 6.7–11.1% were observed [17, 18]. In these studies, TRA failure wasusually followed by crossover to TFA, which is associated with more complications, as has been proven. In our all-comers study population, primary TFA was performed in 1.8% [95% CI: 1.1, 2.6%] of cases. In some cases, reasoning for primary TFA was not adequately documented or seemed to be performed prematurely. This potentially implicates that TFA could have been avoided in some instances and the TFA rate could have been even lower. Nonetheless, TFA was less frequently reported compared to renowned, retrospective trials which reported primary TFA in 23–62% of cases in their respective study population [10, 15, 19]. In our study population, crossover to TFA after one or two failed FAA attempts was observed in 2.8% [95% CI: 1.8 to 3.7] of cases. The observed crossover rate was therefore significantly lower than crossover rates reported by comparable studies, ranging from 4.7–6.9%9, [15, 16.] When primary FAA failed, crossover to TFA could be avoided in 81% of cases due to up to two further FAA attempts. Yet again, despite recise documentation of reasons for FAA failure in general, reasoning for crossover to TFA after one or two failed FAA attempts, was not always sufficiently traceable to justify crossover to TFA. We therefore suppose that crossover to TFA might have been undertaken prematurely in some instances due to operator preference. Hence, it is possible that the crossover rate to TFA could have been even lower if operators had fully complied to our institutional “forearm-only-strategy”. Yet, interventional cardiology with its emergency procedures, complex interventions and time constraints does not always offer the framework to meticulously follow internal policies in daily clinical practice.

We suppose that our relatively low crossover rate is due to our forearm-only-strategy and a decrease in certain etiologies of FAA failure. Regarding the etiologies of FAA failure, we observed a markedly different allocation of reasons for failure compared to previous studies. While other trials reported almost half of TRA failures occurring due to failed arterial cannulation or sheath insertion and significant numbers of arterial spasm necessitating crossover, kinking only represented a small share of all reasons for TRA failure [10, 20]. In our study population, despite still being responsible for more than one third of FAA failures, access site-related FAA failures were less frequent. We suppose that routine ultrasound-guided arterial cannulation might be a potential explanation for this observation since previous studies with relatively high access site-related TRA failure rates did not report routine ultrasound use for arterial cannulation. The superiority of ultrasound-guided arterial cannulation over palpitation-guided arterial cannulation could already be shown in earlier studies [21]. Kinking, at the level of the forearm and the brachiocephalic artery, was the most frequent cause of FAA failure in our study population, being responsible for more than a third of FAA failures and occurring in 3.0% of all FAA attempts. In the beginnings of TRA cardiac catheterization, kinking, especially at the left subclavian artery was estimated to prevent cardiac catheterization in up to 10.8% of individuals [22]. The fact that kinking was the most frequent cause of FAA failure in our study population necessitates further scientific effort to get a deeper understanding of this etiology. When cardiac catheterization was first started to be performed via the radial artery, arterial spasm posed a major problem, being the reason for TRA failure in up to 10.3–14.3% of cases, with some studies reporting rates of up to 34% [23–25]. In our cohort, FAA failure due to arterial spasm was reported in only one (0.08%) out of 1225 FAA attempts. We suppose that this particularly low incidence can be attributed to controlled ultrasound-guided arterial cannulation, causing less irritation to arterial smooth muscles, and the routine use of SFA. When forearm vascular anatomy was assessed adequately via SFA, before guidewire advancement, accidental intubation of forearm artery side branches potentially triggering arterial spasms could be prevented. However, it must be considered that many studies, which reported noticeably higher prevalences of arterial spasm, did not invariably report the routine use of intraarterial vasodilators after sheath insertion. Another subject of our study was kinking, specifically at the level of the forearm. 1st degree kinking at the level of the forearm occurred in 8.0% of cases, while 2nd degree kinking was observed in 1.8% of cases. Especially with 1st degree kinking, SFA has proved its usefulness. While previous studies reported the use of FA only if difficulties with guidewire or guide catheter advancement were noted, we declare ourselves in favor of preventive SFA. We suggest that gaining knowledge about vascular variants at the level of the forearm before carefully engaging the respective section of the vessel with a guidewire is valuable, since manipulation of an inadvertently kinked wire can cause arterial damage or arterial spasm. In our study, kinking at the level of the forearm caused FAA failure in 1.8% of procedures despite SFA and a subsequent change of devices. Interestingly, 1st or 2nd degree kinking were always reported with TRA but never with TUA. Forearm artery vascular complications occurred rarely, causing FAA failure in a total of three cases (two dissections, one perforation). During the study period, not a single case of clinically evident acute hand ischemia was observed. However, patients were not routinely assessed with doppler sonography before discharge. Therefore, postinterventional, asymptomatic arterial occlusion could not be ruled out with our study design. With respect to our angiographic studies, we suppose that forearm vascular supply is abundant due to multiple collateral vessels. We presume that especially the common interosseus artery plays a major role in forearm blood supply preventing symptomatic hand ischemia even in the case of simultaneous radial and ulnar occlusion. We therefore believe that ulnar artery access is feasible, even if the ipsilateral radial artery was harvested for CABG or is occluded due to severe atherosclerosis, since the common interosseus artery provides adequate blood supply to the hand [18] If physiologic forearm vascular supply is confirmed by SFA, subsequent radial and ulnar access on the ipsilateral forearm is presumably safe. However, this was not the aim of our study and therefore we cannot provide scientific evidence to adequately support this thesis. In addition to enabling successful guidewire or guide catheter advancement, SFA also provides essential information about individual forearm vasculature regarding the planning of future procedures. During procedures necessitating a change of the access site, knowledge of the individual forearm vasculature is of fundamental importance for procedural success. Especially in the case of TRA failure, SFA is indispensable to demonstrate an adequately sized common interosseus artery and to foresee vascular variants at the level of the forearm potentially hindering TUA.

## 5. Limitations

Our retrospective analysis is based on procedural data of an intensively trained team of interventional cardiologists and angiologists, who, in the year 2020, set out to avoid the TFA as far as possible. To make a general statement, that SFA decisively increases procedural success rates, randomized controlled multicenter trials are necessary. Due to the ongoing COVID-19 pandemic, case numbers decreased by about 35% compared to the pre-COVID era. However, no substantial differences in baseline characteristics, especially age, sex, or clinical indications, e.g., percentage of patients presenting with STEMI or ACS, were noted.

### 5.1. Impact on daily practice

The 1A recommendation of the ESC for using the transradial-access (TRA)can be implemented in daily routine. This can be reached by integrating standardized forearm-angiography (SFA) in daily practice of the cath lab. In combination with a forearm only strategy the transfemoral-access (TFA) with a higher risk of local bleeding complications can be avoided.

## Figures and Tables

**Figure 2:**
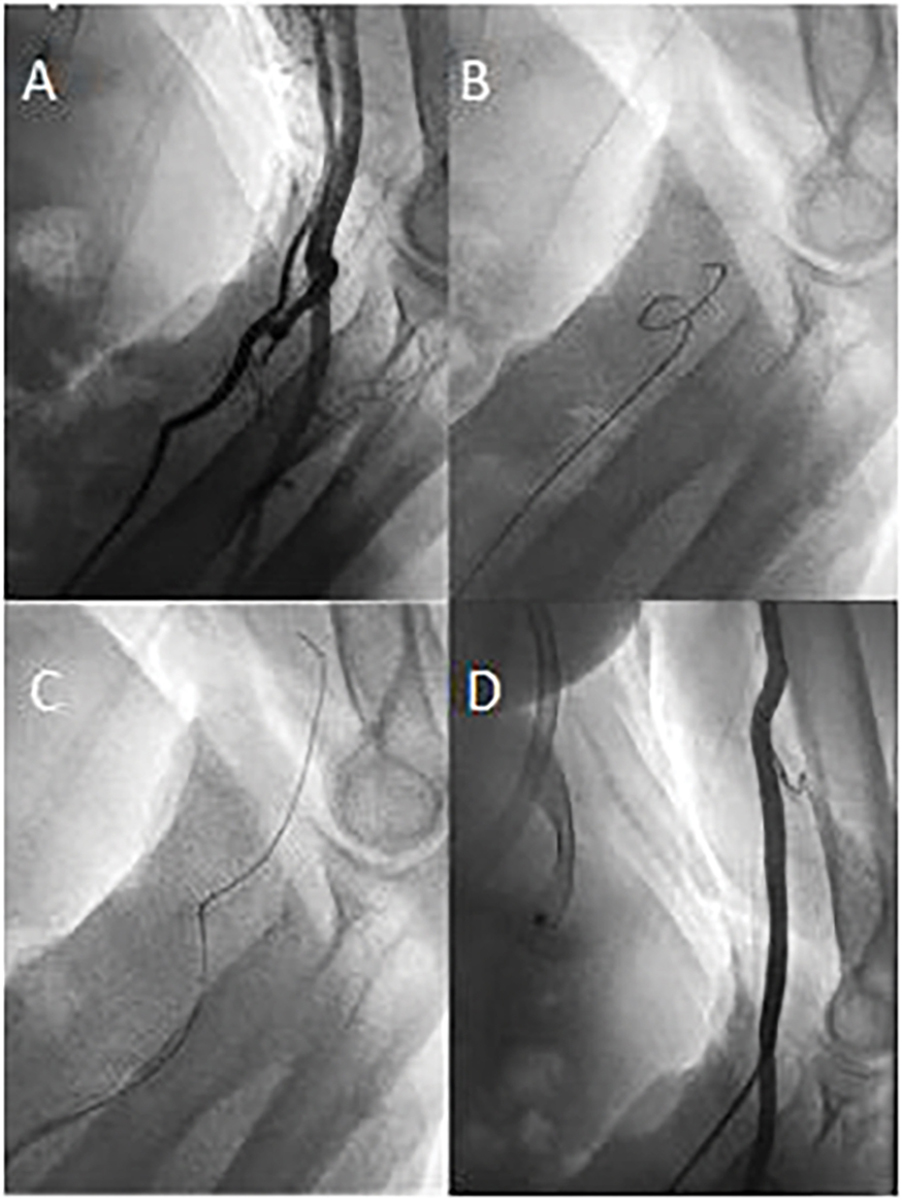
Forearm angiography revealing a radial loop and a collateral radial artery running parallel to the brachial artery **(A)**. The radial loop could initially not be passed with a standard 0.035” J guidewire **(B)** but could be passed successfully after switching to a hydrophilic 0.035” guidewire **(C)** corresponding to first-degree kinking. Subsequent angiography confirmed correct guidecatheter placement in the brachial artery **(D)**.

**Figure 3:**
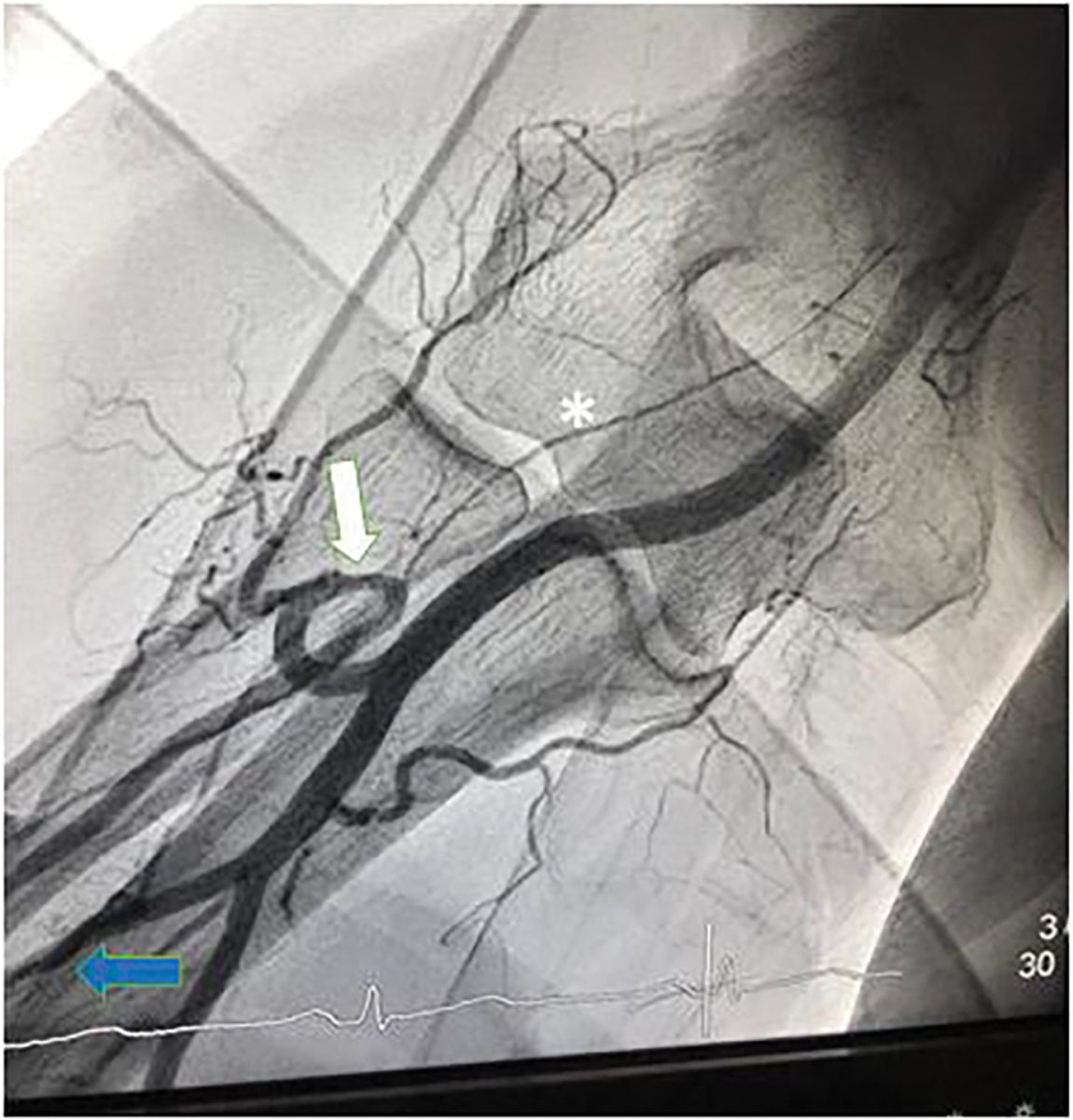
Radial loop (white arrow) with inadvertent intubation of the radial recurrent artery (*). The branching point of the common interosseous artery (blue arrow) from the ulnar artery can be seen at the bottom left.

**Table 1:** 

**Left forearm artery access**	632 (54.2%)
Distal radial artery	408 (64.6%) 5
Proximal radial artery	213 (33.7%)
Ulnar artery	10 (1.6%)
Anterior interosseous artery	1 (0.16%)
**Right forearm artery access**	534 (45.8%)
Distal radial artery	4 (0.7%)
Proximal radial artery	518 (97.0%)
Ulnar artery	12 (2.2%)

Primary forearm access sites (n=121766

**Table 2:** 

Variable	
Age (years)	71.4 [61.0, 79.4]
Weight (kg)	83.0 [72.0, 95.0]
Height (cm)	171.8 ± 9.3
Body Mass Index (kg/m2)	28.4 ± 5.1
Body Surface Area (m2)	2.0 ± 0.2
Female sex	332 (28.5%)
Arterial hypertension	1016 (87.1%)
Diabetes mellitus	325 (27.9%)
Active smoking	294 (25.2%)
Estimated glomerular filtration rate (ml/min/1.73 m2)	74.6 ± 27.9
Percutaneous coronary intervention	475 (40.7%)
Left main stem intervention (7 F)	29 (2.6%)
Coronary bifurcation intervention (7 F)	117 (10.4%)
Bypass graft PCI	2 (0.2%)
Intracoronary imaging and/or physiological assessment	249 (22.1%)
Clinical indication	
Suspected CAD or chronic coronary syndrome	959 (82.2%)
Acute coronary syndrome (ACS)	
ST-Elevation myocardial infarction	81 (6.9%)
Non-ST-Elevation-ACS	121 (10.4%)
Cardiogenic shock	4 (0.3%)
Cardiac arrest	1 (0.1%)

Baseline patient characteristics (n = 1166).

Values are mean ± SD for continuous, normally distributed data, median [IQR] for continuous, non-normally distributed data and n (%) for dichotomous data

**Table 3:** 

Failure to achieve arterial puncture or failure to introduce arterial sheath	32 (34.4%)
Atherosclerotic occlusion	13 (14.0%)
Forearm artery spasm	1 (1.0%)
Periinterventional perforation/dissection of forearm artery	3 (3.2%)
Kinking (brachioradial/subclavian/brachiocephalic)	37 (39.8%)
Failure to intubate coronary ostia	3 (3.2%)
Small target vessel	2 (2.2%)
Other (e.g., following TEVAR)	2 (2.2%)

Etiologies of FAA failure (n=93) in a total of 1225 FAA attempts.

Values are n (%).

## Abbreviations:

ACS: Acute coronary syndrome
CCS: Chronic coronary syndrome
CA: Coronary angiography
ESC: European Society of Cardiology
FAA: Forearm-artery-access
FA: Forearm-artery-angiography
LIMA: Left internal mammary artery
LAO30: 30° Left anterior oblique
TRA: Transradial-access
TFA: Transfemoral-access
TUA: Transulnar-access
SFA: Standardized forearm-angiography
PA: Posterior anterior
PCI: Percutaneous coronary intervention
SD: Standard deviation
IQR: Interquartile ranges
QQ-plot: Quantile-quantile plot
STEMI: ST-elevation myocardial infarction
